# Ethnic identity negotiation among Sami youth living in a majority Sami community in Norway

**DOI:** 10.1080/22423982.2017.1316939

**Published:** 2017-05-03

**Authors:** Kristine Nystad, Anna Rita Spein, Asta Mitkija Balto, Benedicte Ingstad

**Affiliations:** ^a^ Community Medicine and Global Health, Institute of Health and Society, Faculty of Medicine, University of Oslo, Oslo, Norway; ^b^ Center for Sami Health Research, Faculty of Health Sciences, UiT – The Arctic University of Norway, Tromsø, Norway; ^c^ Department of Duodji and Teacher Education, Sámi University of Applied Sciences, Kautokeino, Norway

**Keywords:** Adolescence, ethnic identity, ethnicity, qualitative methods, Sami, health

## Abstract

**Background:** This study was part of the international research project “Circumpolar Indigenous Pathways to Adulthood” (CIPA).

**Objectives:** To explore ethnic identity negotiation, an unexplored theme, among indigenous North Sami youth living in a majority Sami community context in Arctic Norway.

**Methods:** A qualitative design was followed using open-ended, in-depth interviews conducted in 2010 with 22 Sami adolescents aged 13–19 years, all reporting Sami self-identification. Grounded theory, narrative analysis, theories of ethnic identity and ecological perspectives on resilience were applied in order to identify the themes.

**Findings:** All 22 youth reported being open about either their Sami background (86%) and/or ethnic pride (55%). Ethnic pride was reported more often among females (68%) than males (27%). However, a minority of youth (14%) with multi-ethnic parentage, poor Sami language skills, not having been born or raised in the community and with a lack of reindeer husbandry affiliation experienced exclusion by community members as not being affirmed as Sami, and therefore reported stressors like anger, resignation, rejection of their Sami origins and poor well-being. Sami language was most often considered as important for communication (73%), but was also associated with the perception of what it meant to be a Sami (32%) and “traditions” (23%).

**Conclusion:** Ethnic pride seemed to be strong among youth in this majority Sami context. Denial of recognition by one’s own ethnic group did not negatively influence ethnic pride or openness about ones’ ethnic background, but was related to youth experience of intra-ethnic discrimination and poorer well-being. As Sami language was found to be a strong ethnic identity marker, effective language programmes for Norwegian-speaking Sami and newcomers should be provided. Language skills and competence would serve as an inclusive factor and improve students’ well-being and health. Raising awareness about the diversity of Sami identity negotiations among adolescents in teacher training and schools in general should be addressed.

## Introduction

The Sami people are officially known as the indigenous people of Norway. The former assimilation policy known as “Norwegianisation” led to an emphasis on speaking Norwegian rather than Sami, and this policy continued for decades. In the past, many Sami chose to hide their Sami affiliation because of this subjugation [1–3]. As a result of ethno-political movements and renewed understanding of their heritage, Sami now more often see themselves as successful fighters for equal rights, and the feelings of shame and subjugation are now being replaced by a sense of pride [1]. Intra-generational ethnic mobility has maintained the Sami sense of identity, as ethnic affiliation reporting has changed over time from parents claiming no Sami affiliation to their descendants reporting affiliation [4]. Sami youth in Norway grow up in a variety of different ethnic or cultural contexts – some in regions where Sami are the numeric majority, while in others they are the minority. Ethnic mobility has also had an impact on where we find young Sami today. Today’s changing conditions for being Sami in Norway have led to a reversal from feelings of subjugation and shame to cultural pride, as more people claim and express their Sami identity (e.g. [4,5]). Bjørklund describes the political upheavals that resulted from the “Alta case” in the 1980s as an ethno-political earthquake [5]. This ethno-political movement is anchored on the understanding of Sami as something of value from the past and from their ancestors, and this treasure has to revitalise and rebuild Sami society [6]. The overall aim of the study is to investigate Sami adolescents’ pathways to adulthood. In this article, we explore what influences their acceptance as Sami in a Sami-majority community, and how non-acceptance can influence their pathways to adulthood.

Fishman [7] emphasises language as an important marker of cultural identity and argues that ethnicity is “being”, “doing” and “knowing”. “Being” is essential and represents one’s ancestors and heritage, while “doing” and “knowing” are negotiable. Fishman further argues that “doing” and “knowing” are impossible in any language other than the authentic one and that “doings” are *expressible only within traditional networks* [7]. Phinney and Ong [8] emphasise that “…the core of ethnic identity is a sense of self as a group member that develops over time through an active process of investigation, learning, and commitment.” Exploration is essential in ethnic identity formation by seeking information and experiences relevant to one’s ethnicity, which most commonly occurs during adolescence. The study of identity formation addresses the interplay of development pressures and cultural factors [9]. Among ethnic minorities, ethnic pride serves as recognition of one’s ethnic identity [10]. Due to globalisation and modern media technology, young people, including those in rural areas, develop a global identity [11]. This is a positive aspect for those who are not recognised as Sami, as it gives an alternative sense of identity and inclusion. For those with a strong ethnic identity, this can be a very positive way to affirm their sense of belonging to their community and to the wider world.

Strong connection to one’s ethnic group is argued by Wong, Eccles and Sameroff [12] as the principal component of ethnic identity. Moreover, Shelton and colleagues [13] argue that ethnic identity plays an important role in ethnic minorities’ psychological development, and ethnic identity may serve as a buffer against discrimination and general stress [14]. The significance between ethnic identity and resilience is emphasised in an earlier study of indigenous circumpolar youth [15]. Ethnic affirmation and ethnic pride/openness were found to be individual-level resilience factors. Resilience is understood as a dynamic process that contributes to positive outcomes despite exposure to significant adversity [16]. Adolescents’ fundamental sources of resilience are their personal abilities and the cognitive strategies they use to find a healthy pathway to adulthood by using the available resources so as to negotiate and navigate through adversities [17]. Quantitative studies on Sami youth and health in Norway have shown that if majority-Sami communities can encourage attachment to the group, this can lead to fewer behavioural problems [18]. Sami adolescents living in majority-Norwegian contexts reported more behavioural problems than their regional non-Sami peers. Earlier research has revealed that traditional knowledge, cultural practices and activities promote ethnic pride among Sami tenth graders [17]. Further, reindeer-herding affiliation and Sami language competence were regarded as strong Sami ethnic identity markers [19–21].

Sami self-identification was found to depend on the ethnic community context. The 15- to 19-year-old adolescents living in majority-Sami contexts were more likely to report Sami self-identification than peers living in assimilated Norwegian contexts. Those Sami living in assimilated Norwegian contexts were more likely to claim Norwegian national identity [20]. With regards to ethno-cultural factors and drug use, Sami youth living in assimilated Norwegian contexts reported more drinking and intoxication than Sami peers living in Sami-dominated contexts. In addition, Sami youth who thought assimilation was important reported more smoking and drinking than Sami youth who were less supportive of assimilation [22]. Strong Sami ethnic identity in mid-adolescence was associated with less binge drinking in late adolescence and early adulthood. Multi-ethnic parentage, where one parent is Sami with poor Sami language fluency and lacking the ability to speak Sami with friends was also associated with more drug use, but this aspect lost statistical significance when adjusted for other ethno-cultural and socio-economic factors [22]. There is a need for further research on how Sami youth explore their ethnic identities and how ethnic identity formation can contribute positively or negatively to their well-being and health. This article is based on a qualitative study of youth in a Sami-majority community in inner Finnmark and addresses ethnic identity negotiations among young Sami. However, there are a few studies using a qualitative approach (e.g. [6,23]).

## Aims and objectives

The overall aim of the study was to explore what it means to be Sami in a group of young Sami who identified themselves as Sami during mid and late adolescence. The research questions were:

- What are the ethnic markers associated with Sami self-identification among Sami youth living in a majority-Sami context?
- What are the significant ethnic or cultural markers that determine the recognition or denial of their Sami ethnic identity in this Sami-majority study community?
- How does the denial of Sami ethnic identity impact the youth’s well-being, health and future plans?

## Participants and methods

### Participant recruitment

A total of 180 students in the community were invited to participate. This included students in lower (eighth to tenth grade) and upper secondary school (high school). Both verbal and written information (brochures and letters) concerning the study, as well as a consent form for permission to participate in the study, were given to these students. In addition, information about the study was broadcast by the local radio station and advertised in the newspaper. The first 22 students who signed up were asked to recruit others through snowball sampling of friends. This resulted in others joining the survey, including one person who had dropped out of school. Of the participants, 11 had reindeer herding affiliation. All participants reported Sami self-identification.

### Methodological approaches

This study, conducted in 2010, was a part of an international study on Circumpolar Indigenous Pathways to Adulthood (CIPA) conducted in five circumpolar sites in the USA (two Alaskan sites), Canada, Russia and Norway [24]. The collaboration with the international research team helped to identify common themes across the sites. These included the challenges faced by the young people and possible resilience strategies and potential community resilience factors. All study sites utilised the same basic research design and methods, and the interview protocol and development process has been described elsewhere [24,25]. However, a specific research protocol to reflect the local context of the Sami study community was conducted in collaboration with a local steering committee composed of politicians, a health care professional, a school social worker, co-authors, an elder and two youth. The steering committee assisted in both the recruitment and interpretation of data. Qualitative methods such as semi-structured interviews were chosen in order to explore the challenges that the young people were facing and the social, symbolic and material resources that they were using in their pathways to adulthood [24].

### Qualitative interviews

The semi-structured interview guide included open-ended questions on the following themes: school, Sami culture and language, family, perception of youth in the community, relationships, health, their future hopes and plans and how they had overcome any challenges they had faced (see Appendix). The points of interest for this article were their ethnic identity formation, how they handled their ethnic identity in a Norwegian context and what identifies them as Sami. All interviews were conducted by the first author and took place in the schools during the school day. The interviews were conducted over two sessions, which lasted between 30 and 60 minutes. The second session allowed the researcher to conclude the interviews and to develop further responses to the questions asked in the first session. Five of the participants were interviewed in Norwegian as they lacked Sami language competence or had Norwegian as their primary language. Interviews were recorded and transcribed by the first author and translated into English by a translator who had cross-cultural competence in Sámi culture and reindeer husbandry. The Norwegian transcripts were translated by two other translators. The translations were reviewed by the first author to ensure accuracy and reliability.

### Analysis

The data were analysed by using grounded theory analysis. Transcripts were uploaded to Altas.ti software and the software helped to identify relationships between coded categories. Initial coding was followed by selective coding and the most significant and/or frequent codes surfaced, followed by decisions about which codes made the most analytic sense in order to categorise the data [26]. For instance, the ways in which the adolescents handled their ethnic identity, language and culture in non-Sami settings were coded as “Ethnic openness” or “Ethnic pride”. Memos helped to capture comparisons and connections between data and codes and led to new ideas.

In addition to grounded theory analysis, we wrote brief narratives [27] that provided a case-centered interpretation of the data and described the challenges that the young people were facing and how they were negotiating and navigating through these adverse conditions. Theories of ethnic identity development and significance for minorities, as well as ecological perspectives of resilience, helped to identify these themes [7,28].

### Ethics

This study received prior approval from the Norwegian Regional Committees for Medical and Health Research Ethics (REC) 2009/729-2. Voluntary participation was emphasised and, during the study, participants were given the option to withdraw at any time. For those who volunteered to participate and were younger than 16 years of age (the age of consent in Norway), informed consent was obtained from parents or guardians. The study touched on sensitive matters, making it important to ensure informant anonymity in order to protect privacy. As a part of the community and having knowledge about the community, the first author had to consider which information should be used and how this should be presented. Being aware of accepted research and ethical guidelines, we had to pay attention to the young participants’ limits and needs.

## Findings

The findings of adolescent ethnic identity negotiation and navigation on their pathways to adulthood are based on a grounded theory analysis of the transcripts, selected quotations and brief narratives. Table 1 outlines the research themes and questions concerning the Sami language and culture and findings related to ethnic identity and ethnic markers. It represents adolescents’ ethnic parentage (mono- vs. multi-ethnic Sami), fluency in the Sami language (yes vs. no), mother tongue (Sami vs. Norwegian) and attitudes towards the Sami language and how they would handle their Sami identity in a Norwegian context. The participants represent two main categories: those (86%) who experienced Sami ethnic identity affirmation and those (14%) who experienced intra-ethnic exclusion or discrimination. Table 2 presents three narratives representing different strategies for dealing with Sami ethnic identity negotiation and navigation.

**Table 1.** Sami language and culture themes: coding of ethnic markers among 13–19-year-old Sami adolescents in a Sami-dominant community.

Informant (number)/gender (M, F)	Sami parentage (mono- vs. multi-ethnic)	Sami mother tongue (yes vs. no)	Research questions: *What does being Sami mean to you?* *Do you feel like a Sami?* *Do you speak the language?* *What does it mean to you to be able to speak Sami?* *What is important to you about your traditional culture?*	Coded as: communication, ethnic identity marker, cultural bearer, place attachment, reindeer husbandry affiliation	Research questions: *When you go to Oslo (Tromsø, Hammerfest) or to other places outside Sápmi, do you tell people you are a Sami?* *Have you sometimes been ashamed of being a Sami?* *Have you sometimes been proud of being a Sami?*	Coded as: ethnic pride, ethnic openness, traditional costume
1, F^1,2^	Mono-ethnic	“Yes	“It means a lot to me. We don’t speak Sami at school because one of our friends is Norwegian speaking. It’s a pity not being able to speak our language.”	Communication	“…if people ask, I would tell them, and not try to hide my Sami background…”	Ethnic openness
				Cultural bearer/ traditions		
2, F^2^	Multi-ethnic	No	“Sami means a lot really. First and foremost a language you speak and there’s a lot of history behind the language. Young people and adults in general must be able to speak Sami, and it’s almost a struggle to fit in for everybody.”^6^	Communication	“I am proud of being Sami…because the Sami are an indigenous people…”	Ethnic pride
				Cultural bearer/ traditions		
3, F^1,2^	Mono-ethnic	Yes	“The Sami language is central here in our village as well as in reindeer herding. And it’s also the mother tongue for most people here. If I lived outside Kautokeino maybe I wouldn’t care much about Sami language.”	Communication	“I’m not embarrassed about being a Sami, but I don’t walk around telling everyone I’m Sami. If they ask, I will let them know I am Sami if they ask me.”	Ethnic openness
				Place attachment		
				Reindeer husbandry		
4, F^2^	Multi-ethnic	No	“I try to learn Sami. I still think it was a pity that we lost the language. Otherwise I could have actually been able to speak Sami fluently.”^6^	Communication Place attachment	“…If they ask if I’m Sami I tell them of course, I don’t keep it a secret… I’ve been proud of being Sami very often.”	Ethnic openness Ethnic pride
			What does it mean to you being able to speak Sami? “A lot while you are living here in this place. Be able to understand. There are many people who don’t understand Norwegian here. It would have been easier to talk to them if I knew Sami.”			
			“You don’t need to speak Sami to be a Sami.”^6^			
5, F^1,2^	Mono-ethnic	Yes	“Sami language means a lot to me. It is my mother tongue and I speak it every day. It would be sad if I lost my language. Of course people must speak the Sami language when they are Sami!”	Communication	“I’m not ashamed of being a Sami! People can think whatever they want but I’m not ashamed of my culture! I don’t hide my identity. I’m very proud when I’m wearing our traditional regalia, then I get lot of attention.”	Ethnic openness
				Ethnic identity marker		Ethnic pride
						Traditional costume
6, F^2^	Mono-ethnic	Yes	“Sami is the language I speak every day. I like to speak Sami much more than Norwegian, but I’m better at writing Norwegian than Sami.”	Communication	“I don’t ever try to hide my Sami background. Last year I was proud of having the opportunity to tell other adolescents about the Sami as they wanted to know more about the Sami.”	Ethnic openness
						Ethnic pride
7, F^1,2^	Multi-ethnic	Yes	“It doesn’t mean anything special but it’s good to know the language.”	Communication	“I’d tell people I’m Sami. I have been proud, especially when we put on the Sami clothes going to a non-Sami place. It feels good to be a Sami. Especially when the people there haven’t seen Sami people before. They like to take pictures. They ask stuff like: ‘Do you really have your own reindeer?’ They don’t know. They think we live in a *lavvu* [Sami tent] all the time. We tell them stuff that they don’t know and they’re very surprised.”	Ethnic openness
						Ethnic pride
						Traditional costume
8, F^1,2^	Mono-ethnic	Yes	“It’s very important to me. I think it’s good to have our own language. Norwegian is the main language but then we have Sami too.”	Communication	“I tell them that I’m a Sami and I try to show them too. When we were in Canada people were very fond of us. …They wanted to exchange jackets with us and even want to sell a jacket to us. …They also asked a lot about us.”	Ethnic openness
						Ethnic pride
9, M^2^	Multi-ethnic	No	“Sami is a language that doesn’t belong to any country, a language for Sami who don’t have their own country. I’m not good at that language which is bad as of course I’m Sami myself. It’s a challenge. I’m not accepted as Sami. I’m assumed to be Norwegian. Quite okay for me.”	Ethnic identity marker	“Yes, I would do that. I’m not embarrassed about being Sami. I’m actually proud of it.”	Ethnic openness
						Ethnic pride
10, M^2^	Multi-ethnic	No	“It means…I don’t know what it means. It is of course nice. Sami is good. Sami is my favourite subject. That isn’t difficult.”	–	“I say something if I’ve got to know someone, then I tell them. I’m not afraid to say that I’m Sami. When they found out where I lived then they would know I’m Sami. They couldn’t hear it from my dialect since I don’t speak Kauto dialect.”	Ethnic openness
11, M^1,3^	Mono-ethnic	Yes	“A Sami speaks Sami.”	Ethnic identity marker	“I wouldn’t tell anyone that I’m a Sami. One never knows what mad person you might meet. There are racists…I haven’t experienced racism other than on Nettby [Internet]. When some people see a picture of persons wearing the Sami regalia they have written some comments of a racist character.”	Hiding or lack of ethnic openness of his Sami background due to experience of discrimination and prejudice
12, M^1,3^	Mono-ethnic	Yes	“Nothing in particular.”^6^	–	“I would say I’m Sami. Not hide it, not at all…”	Ethnic openness
13, M^1,3^	Mono-ethnic	Yes	“Everyone speaks Sami here. If you don’t know the Sami language then you won’t understand what they are talking about. It would be tiresome to speak Norwegian when all the others are speaking Sami.”	Communication	“Yes, I can tell them about my origin if they ask me. Do you try to hide your identity?” “No, I don’t hide…what is there to hide? I’m not ashamed of being a Sami.”	Ethnic openness Ethnic pride
14, F^1,3^	Multi-ethnic	Yes	“In my opinion it’s necessary to have the Sami language in reindeer husbandry as well as in other cultural Sami traditions because there are words that explain things in a way that no other language ever will.”^6^	Communication	“I wouldn’t hide my Sami identity. I would let everyone know who wants to…In big gatherings I’ve been very proud of being Sami.”	Ethnic openness
				Reindeer husbandry		Ethnic pride
				Cultural bearer		
15, M^1,3^	Mono-ethnic	Yes	“The language all Sami speak.”	Ethnic identity marker	“I’m not afraid of telling people I’m Sami.”	Ethnic openness
16, M^1,3^	Mono-ethnic	Yes	“Sami is very important and gives you a lot of opportunities. It’s the language I’ve been brought up with. If Sami didn’t exist, it would be strange. It’s very important.”	Communication	“I don’t write on my forehead that I’m Sami. …I don’t try to hide it. Are your proud of it? Yes I am…In big gatherings when I am wearing ‘*gákti*’^4^ Then I feel much richer: I have more than one culture and languages.”	Ethnic openness
						Ethnic pride
						Traditional costume
17, F^3,^^5^	Mono-ethnic	No	“Eh…richness in a way. …Often when they are searching for a new staff member, they want someone who knows Sami. It’s good to have on your CV. It’s the language I speak daily since I learnt it.”	Communication	“It depends on who’s asking…I don’t walk around wearing my ‘*gákti*’^4^ and telling everyone I’m a Sami. However if people ask, and know I’m from the study community, I don’t want to hide it. If someone asks, I’ll tell them I’m Sami.”	Ethnic openness
21, M^2^	Mono-ethnic	Yes	“That’s the language we talk now. A language is important and if we stop using it and start speaking Norwegian instead, then the Sami language will die and that would be sad.”	Communication	“I’ll tell people I’m Sami…I would only be identified by my accent, as we wear similar clothing [to Norwegians] in everyday settings.”	Ethnic openness
				Cultural bearer/traditions		
22, M^2^	Mono-ethnic	Yes	“If I’m in a city and speak Sami, they’ll identify me as Sami.”	Ethnic identity marker	“When I’m wearing a ‘*gákti*’^4^ then people outside the community will identify me as Sami.”	Ethnic openness
						Traditional costume
23, F ^2^	Multi-ethnic	No	“I could speak Sami ever since I was little. I learned both languages just as well. My mum and dad speak Norwegian at home all the time. I have Norwegian at home and I speak Norwegian with my brothers. At school I speak Sami and with my grandmother.”	Communication	“I don’t hide my Sami background and others will realise I’m Sami as I often mix the Sami and Norwegian languages.”	Ethnic openness
24, M^2^	Mono-ethnic	Yes	“It a language I speak normally. They will identify me as Sami by the language I speak.”	Communication	“I don’t walk around telling people I’m a Sami, but if they ask I tell them I’m Sami. They may also identify me as Sami by my language.”	Ethnic openness
				Ethnic identity marker		
25, M^1,2^	Mono-ethnic	Yes	“It’s important to know the Sami language. I’m not very good at writing… I’m fluent in both languages [Sami and Norwegian]. …People usually hear from my dialect that I’m from the northern part of Norway.”	Communication Ethnic identity marker	“They would know by my acts. For example if I went out on the land, they would be able to see that I know how to look after myself there.”^6^	Ethnic openness

F: female; M: male.

^1^ Reindeer husbandry affiliation.

^2^ Lower secondary grades: 13–15 years.

^3^ Upper secondary grades: 16–19 years.

^4^ Sami clothing.

^5^ A school dropout.

^6^ Quotes or findings have previously been published [21].

**Table 2.** Sami ethnic identity negotiations: adolescents’ narratives of ethnic identity denial (three participants).

Informant/gender	2, F	4, F	9, M
Ethnic parentage	Multi-ethnic	Multi-ethnic	Multi-ethnic
Language skills in terms of mother tongue and first language	Non-Sami mother tongue, limited Sami language skills	Non-Sami mother tongue, limited Sami language skills	Non-Sami mother tongue, limited Sami language skills
Place attachment	Newcomer. Lived for a period in the community in her early childhood. Moved back recently	Newcomer in the community	Lived in the community permanently (parents were newcomers)
Narrative	“Anne” and her family had lived in the community earlier and the reason for returning to the community was bullying at the school she attended in the city. She states that she was bullied because of her Sami background (in a Norwegian-dominant area). After she moved to the community, she tried to learn the Sami language. It is difficult for her to understand what the other students are talking about in the class, since they speak Sami. She often feels that she does not fit into the community because she does not speak the language. In her opinion, people have to be able to speak the Sami language in the community in order to fit in. Her main stressor is that she does not feel that she fits into the community and she plans to leave the community in the future	“Nina” was raised in a coastal Sami area where Sami was in the minority and many families have hidden their Sami identity. She was not aware of her Sami ancestors before she moved to the community. Nina has tried to learn to speak Sami and, in her opinion, it was a pity that her family had lost their Sami language during the assimilation period. Based on her own experience, Nina feels that non-Sami have a negative opinion about Sami. Nevertheless, she is proud of her background, but is stressed about not being recognised as Sami by her peers and other community members. She gets annoyed because people tell her that she is not Sami. She argues that she has Sami ancestors, wears a “*gákti*”^1^ and feels Sami, and she thinks that you do not need to speak Sami to be a Sami^2^	“John” is stressed about not being accepted as Sami by his peers. He is struggling to learn Sami and feels it is a pity not to be able to talk Sami as he is Sami himself. He feels that, in order to live in the community in the future, knowledge of Sami culture would be essential. When talking about what things he has learned that he could use later in life, he said: “Everything, but not Sami. I don’t plan on living in a Sami community, so actually Sami doesn’t mean anything. I don’t want to be Sami. I can be Sami if I want, but I’m not going to use the language later when I finish secondary school. I’m going to go to high school where the language of instruction is Norwegian. When I finish there I’m going to try to get a house in Asia.”
Strategy	Resigning to non-acceptance	Negotiating her own Sami identity	Escaping from his Sami background

F: female; M: male.

^1^ Sami traditional costume.

^2^ Quotes previously published [21].

### Sami language as an ethnic identity marker

As seen in Table 1, the Sami language is not only considered highly valuable for communication (73%), but is also an ethnic identity marker (32%) and an important part of traditions and reindeer husbandry (23%). Most respondents (77%) had Sami as their mother tongue and spoke it daily. Sami language was considered to be an ethnic marker, and as stated by one participant [11], “A Sami speaks Sami.” Another participant [15] confirmed that “Sami is the language all Sami speak.” In Norwegian contexts, the Sami language might be the factor that identified the adolescents as Sami, as one male participant [22] said: “If I’m in a city and speak Sami, they’ll identify me as Sami.”

Participant 2, a newcomer in the community with multi-ethnic parentage who lacked Sami language competence, said that one cannot fit in without speaking Sami. Another newcomer [4] realised that it was necessary to speak Sami in the community in order to understand and communicate with people. She felt anger at the loss of the Sami language in her family during the Norwegianisation period: “I still think it was a pity we lost the language. Otherwise I could have actually been able to speak Sami fluently.” She argued that one can be Sami without speaking the language.

### Place attachment and cultural practices as ethnic identity markers

For 27% of the adolescents, language was closely related to place attachment and cultural practices such as Sami traditional knowledge and reindeer husbandry. Outside the community, as stated by one female participant [3], the Sami language would not be so important. In reindeer husbandry and Sami cultural traditions, the Sami language was considered necessary: “In my opinion it’s necessary to have the Sami language in reindeer husbandry as well as in other cultural Sami traditions because there are words that explain things in a way that no other language ever will.”^1^ ^1^This quote has previously been published [21].

### Ethnic pride and ethnic openness

All participants self-identified as Sami (n=22), and the majority expressed ethnic openness (86%) and/or ethnic pride (45%) when being in a majority-Norwegian context (Table 1). However, one male would hide his Sami background for fear of discrimination and prejudice that he had experienced on the Internet. The majority of the adolescents were open about their Sami ancestry when explicitly asked. Some adolescents reported that they were identified as Sami by their Sami language, Norwegian accent and ethnic symbols such as the use of traditional costume. Ethnic pride was more often expressed by females, as 68% of females and 27% of males reported ethnic pride. Although reindeer husbandry is considered a “cultural bearer” of Sami-ness, only 6 of the 11 (54%) reindeer husbandry-affiliated adolescents reported ethnic pride: four females and two males. Four of the five with multi-ethnic parentage reported ethnic pride (Table 1).

### Sami ethnic identity negotiations

Those who reported struggling most with their ethnic identity were in the youngest age group (13–15 years), had multi-ethnic parentage, did not have Sami as their mother tongue, had limited fluency in Sami and had weak place attachment (Table 2). The three narratives in Table 2 (participants 2, 4 and 9) outline their negotiations and navigations through their sense of identity. The narratives also represent different feelings and strategies regarding experiences of intra-ethnic exclusion. By contrast, multi-ethnic Sami youth who felt included by peers had reindeer husbandry affiliation, had been raised in the community and had an extended family living within the group. “Anne” [2] had experienced bullying in Norwegian contexts because of her Sami background; however, in the Sami community, she also felt that people denied her Sami identity. “Nina” [4] had found that people in Norwegian contexts generally misunderstood and held negative opinions about Sami, calling them people of the devil. “Nina” had also experienced Sami language loss in her family. She explained that it is upsetting for her when people say she is not Sami; she argues that she has Sami ancestors and she wears the Sami clothing “*gákti*”. “John” [9] also felt that he was not accepted as Sami and had developed ambivalence towards his Sami background. Although he expressed Sami pride, being Sami meant little to him (Table 2).

### Health

Health was mainly considered as eating healthy food and being fit. One female informant [14] referred to health as “Being fit and eating nutritious food.” Another believed, “A person’s healthy when they don’t have any major problems like cancer or heart diseases. Smaller health problems is something everyone has, I’m surely not the only one assuming that.” However, two participants seemed to view health as including mental health: “I mean not having any diseases or disabilities that prevent you from thinking normally.” Another stated, “I think it’s important to think in the right way and that your body functions correctly.”

## Discussion

The findings revealed that Sami identity recognition was related to ethnic markers such as ethnic parentage, Sami language competence, place attachment, reindeer husbandry affiliation and/or use of ethnic symbols. A summary of these ethnic identification factors is shown in Tables 3 and 4. Adolescents (n=3) who were denied their ethnic identity by peers were mainly Norwegian speaking, had multi-ethnic parentage and weak place attachment and reported to some degree knowledge about the Sami culture (Table 3), while their peers reported traditional knowledge and were able to survive on the land.

**Table 3.** Summary of reported Sami ethnic markers comparing those who felt that their Sami ethnic identity was affirmed with those who felt that their Sami ethnic identity was denied (n=22).

Ethnic markers	Characteristics of adolescents who experienced ethnic identity affirmation (n=19, 86%)	Characteristics of adolescents who experienced ethnic identity denial (n=3, 14%)
Ethnic parentage	Predominately mono-ethnic (79%)	All reporting multi-ethnic Sami parentage (100%)
	Multi-ethnic Sami parentage (21%)	
Sami language	Sami as mother tongue or, to some degree, Sami language skills (100%)	Norwegian as first language and poorer skills in Sami language (100%)
Ethnic openness	100%	33%
Ethnic symbols (Sami clothes)	21%	33%
Place attachment (born and/or raised in the community)	100%	33%
Extended family	100%	33%
Reindeer husbandry affiliations	50%	0%
Ethnic pride	Less than half (32%)	100%

**Table 4.** Summary of findings: distribution of ethnic markers among indigenous Sami youth by ethnic parentage and ethnic identity (n=22).

	Ethnic parentage		
	Mono-ethnic (n=15)	Multi-ethnic (n=7)	
	Ethnic identity affirmation (n=15)	Ethnic identity affirmation (n=4)	Ethnic identity denial (n=3)
Sami language fluency	100%	50%	0
Sami language as:			
– Communication	73%	75%	67%
– Ethnic identity marker^1^	86%	0%	33%
– “Traditions”	60%	20%	33%
Ethnic pride	33%	50%	100%
Ethnic openness	100%	75%	67%
Reindeer husbandry affiliation	60%	50%	0%
Extended family^2^	100%	100%	33%^3^
Ethnic symbols^4^	20%	25%	33%^5^

^1^ Reporting what it meant to be a Sami.

^2^ Reported in Table 2 by Informant 2, “Anne”.

^3^ Extended family either through mother or father.

^4^ Use of Sami traditional clothing.

^5^ Reported in Table 2 by Informant 4, “Nina”.

### A positive sense of ethnic identity and ethnic pride

The vast majority of adolescents felt that their ethnic identity was affirmed. They generally expressed ethnic pride and were open about their Sami affinity in Norwegian contexts. Hiding one’s ethnic affiliation was caused by experiencing discrimination. Sami adolescents in this study are more open about their Sami affinity compared to earlier findings [29] in which monolingual Norwegian-speaking adolescents were found to be ambivalent about expressing their Sami affinity, and were especially reluctant to do so in Norwegian contexts. This represents an ethnic reversal from shame and hiding Sami affinity [1] to ethnic openness and pride. Adolescents not recognised as Sami and unable to speak Sami also expressed ethnic pride. Even when taking into account the small number of study participants, these responses indicate that a lack of recognition and language competence in a Sami context does not negate a feeling of ethnic pride. Multi-ethnic parentage was generally not a negative aspect of being recognised as a Sami; adolescents with a reindeer husbandry affiliation, Sami language and extended family did not struggle for Sami recognition. Ethnic context, mother tongue and livelihood (reindeer husbandry) have previously been found to positively influence Sami identity [21].

The findings indicated a gender difference with regards to ethnic pride; girls aged 13–15 years were more likely to express their ethnic pride than males. This is in line with research that has revealed that parents emphasise ethnic pride in the socialisation of girls [30]. While this can be seen as an example of resilience, the study also showed that is can be a risk factor for girls when the group is being discriminated against. Lack of openness about one’s ethnicity was due to inter-ethnic discrimination [31]. This inter-ethnic discrimination can be within the society where youth experience racism or in the broader global village due to negative comments and stereotypes about ethnic origin and affiliation.

### Sami language as key for participation and as an ethnic marker

Sami language was considered to be a vital ethnic marker in the community, indicating that those who could not speak Sami felt that they did not fit in. According to Barth [32], ethnicity is an outcome of social interaction. Poor fluency in Sami might hinder participation in social interaction in various community networks. Only the authentic language – in this case Sami – can be used in traditional ethnic networks [7], such as reindeer husbandry. Place attachment, such as being brought up in the community or having a family relationship to the community, influenced participants’ inclusion in social networks and thereby their ability to learn the language and cultural practices. Sami cultural competence among Sami youth influences how they are valued by their peers. Åhren [33], in her study of Swedish Sami youth, states that the youth “…who has the largest Sami cultural competence generates a higher value than other Sami and thus they have created a cultural ladder.” The sense of oneself as a group member, which is the core of ethnic identity, develops over time through an active process of investigation, learning and commitment [8].

The two opposing views expressed on language as an ethnic marker were: “You don’t need to speak Sami to be Sami,” and “A Sami speaks Sami.” The Sami language was an ethnic marker in Norwegian contexts, as it was considered that using the Sami language would clearly identify a Sami youth. Being unable to speak Sami while identifying oneself as Sami was associated with negative feelings such as anger and perceived exclusion. Anger was also related to the historical family’s loss of the Sami language due to Norwegianisation. Whitbeck and colleagues [34] have found that historical trauma among Native Americans due to loss of language and culture, in terms of anger/avoidance and anxiety/depression, is salient in the minds of the current generation.

A multi-ethnic family background seems to lead to poorer Sami language skills, which appears to influence recognition in this majority-Sami community [22]. However, this study and earlier studies in both Sami and non-Sami contexts found that Sami language fluency was not crucial for self-identification as being Sami (e.g. [6,35–37]). As such, while studies have revealed that native language competence is essential for a sense of belonging within the ethnic group, for community cohesion and for network participation [21,38,39], not being competent in the language could lead to feelings of alienation and exclusion. As highlighted by others [40,41], our findings indicate that language binds people together, but can also forcefully divide people in indigenous communities.

### Strategies in response to denial of Sami ethnic identity

Three young Sami respond differently to the denial of their Sami-ness: “Anne” intends to resign from the community as a result of not being accepted as Sami. “Nina” has no intention of giving up her Sami identity and intends to negotiate for her place in the community. “John” is ambivalent towards his Sami background. Coming from a multi-ethnic background and having one’s Sami identity denied might lead to resignation and non-acceptance within the study community. Lack of recognition from the Sami group and inter-ethnic discrimination represent a double burden that may result in marginalisation. However, ethnic identity does not operate alone; individuals can have both a national and an ethnic identity [8]. In a majority-Sami context, 48% of Sami adolescents reported having bi-cultural identity, compared to 21% of Sami youth in majority-Norwegian contexts [13].

Another strategy is to fight for recognition and integration by arguing that the Sami language and cultural competence are not crucial for being Sami. Having Sami ancestors and using traditional Sami costume and symbols can also be important factors in having a strong ethnic identity. This can be seen as a “battle” between two different views of being Sami. The “battle” about Sami ethnic identity is conceptualised by Bjørklund [5], who discusses two analytical approaches on Sami-ness: a constructivist and an essentialist approach. According to Giddens [42], in modernity, identity has to be explored and constructed as part of a reflexive process, but in traditional cultures, the identity development between adolescence and adulthood was clearly staked out. The essentialist approach is based on looking at the ethnic and historical reality. The essentialist view of being Sami presupposes cultural competence: Fishman’s “doing” and “knowing” [7]. Bjørklund [5] argues that the constructivist approach to Sami-ness is politically constructed, based on ancestral Sami language competence.

Ambivalence towards one’s Sami heritage and negative attitudes towards Sami caused by low affirmation might lead to a lack of assimilation or marginalisation. Alternatively, identifying oneself with a national or global culture might be an adaptive response to exclusion from one’s own group. Due to globalisation and modern media technology, young people, including rural dwellers, can develop a global identity [11].

Globalisation and the Internet allow young people to choose between a wide range of cultural spheres and communities and thereby become less marginalised. Adolescents excluded from a minority group will experience less stress and more life satisfaction as increasing one’s global identity may have certain advantages over increasing one’s ethnic identity [43]. Weaver [44] describes a similar controversial situation among native North Americans and postulates that identity negotiations will always be based on the use of power and exclusion; someone must be excluded in order for identity to be meaningful.

### Ethnicity and health

Ethnic pride is often considered to be a predictor of well-being and health [45]. Quantitative measures of ethnic identity and acculturation among young Sami in Norway have revealed both negative and positive health impacts [39,46,47]. Sami adolescents with a stronger sense of ethnic identity were less likely to binge drink than peers with a poorer sense of Sami ethnic identity [22]. Findings among Swedish Sami youth revealed that poor treatment due to their Sami ethnicity caused stress [48]. Spein and colleagues [49] found that about 80% of Sami tenth graders in 2003/2004 reported their health as “good” or “very good”, and this was slightly higher in males than females. Good self-reported health was related to physical activity, and poor self-reported health was due to suicidal behaviour. One limitation of the study was the lack of inclusion of cultural factors. The adolescents in the present study associated good health with absence of disease, eating healthy food and exercising, while self-reported health was not addressed.

A quantitative study by Bals and colleagues found that there was a gender difference in relation to ethnic pride in Sami contexts, which was associated with greater externalising problems for girls, but fewer such problems for boys [19]. Ethnic pride is often considered to be a protective factor in affirming a person’s well-being [45]. However, ethnicity also represents vulnerability. Adult Sami with the strongest Sami affiliations reported the highest levels of inter-ethnic discrimination [31]. In a study of European–American and African–American adolescents, individuals with low ethnic affirmation experienced more depression and internalising and externalising problems [45]. Lack of recognition from one’s own ethnic group and inter-ethnic discrimination represent a double burden. Marginalisation can impact health negatively, leading to poor psychological adjustment, substance use, etc. [50]. It is stressful to be rejected by one’s own ethnic group and discriminated against by the dominant group [51]. Bi-culturalism and integration have been found to provide the best health outcomes [50]. Marginalisation and assimilation, combined with a strong ethnic identity, were identified as having negative impacts on Sami adolescents’ mental health [52].

### Limitations and strengths

The recruitment of participants was not limited to school pupils, although 21 were currently in school. The most traditional adolescents might have dropped out of school, which could have influenced the selection of participants. Self-selection bias is a limitation, as participants were allowed to decide entirely by themselves on their participation in the study; thus, they may not adequately represent the adolescents in the community. The findings might have been different if the study had been conducted in a majority-Norwegian context. However, the first author’s knowledge of the Sami language, culture and community was beneficial to identifying themes and interpreting data. The first author’s position could also have been a disadvantage, as certain issues might have been taken for granted, and implicit issues might not have been made explicit. The first author’s ancestral connection to the community might have influenced what the participants shared; some may not have been open in case the information they shared was used against them. Alternatively, some participants might have been more open with the first author due to her cultural knowledge, connection to the community and Sami language fluency.

## Implications

Both school and public health nurses in majority-Sami communities should be aware of the significance of the Sami language for inclusion/exclusion. Furthermore, communication and effective language programmes should be offered in school for language revitalisation. Increased knowledge about Sami youth and ethnic identity management in Sami schools and teacher training programmes is needed. The key to healing communities is to start with individual students, followed by groups of students, and finally by including the entire school in such processes that involve the greater community. This will create healthier and more resilient school environments and community relationships in general.

## Future research

Additional qualitative studies could contribute further knowledge on the influence of intra-ethnic conflicts on Sami adolescents’ ethnic identity exploration and commitment. There is a need for more in-depth research on the consequences of intracultural rejection and intracultural discrimination. When comparing Sami youth living in majority-Norwegian and majority-Sami contexts, the use of both quantitative and qualitative methods, surveys and in-depth interviews would enhance our knowledge of ethnic identity exploration, commitment and acculturation strategies. Longitudinal studies would also be beneficial for studying ethnic identity exploration in adolescence.

## Conclusion

All participants in this majority-Sami context reported Sami self-identification. The vast majority were open about their Sami ethnicity in majority-Norwegian contexts. Girls were more likely to express ethnic pride than boys. Sami youth living in this majority-Sami context who came from a multi-ethnic background, had poor Sami language competence and had weak place attachment felt that their Sami ethnic identity was denied. Even those who felt that their peers rejected their Sami identity expressed ethnic pride. Ethnic pride seems to be unaffected by exclusion or lack of acceptance by one’s own ethnic group. The lack of ethnic openness was due to fear of racism. Sami language competence was considered to be a significant ethnic identity marker. Having one’s Sami identity affirmed in a majority-Sami context was essential for the adolescents. However, having one’s Sami identity denied proved to negatively influence adolescents’ well-being.

As a result of feeling that their Sami identities were denied by their peers, adolescents are finding different pathways to adulthood, such as moving away or continuing to struggle for acceptance within the community. The findings also indicate that the exploration of their national and/or global identity might assist them in achieving a healthy sense of well-being. Sami adolescents are involved in an intra-ethnic discourse/conflict based on constructivist and essentialist perceptions of what it means to be Sami. Contexts that support indigenous Sami culture, such as that of the study community, support adolescents’ attachment to the ethnic group. However, the Sami ethnic identity struggle is also an issue in Sami contexts, although it is not a problem for most adolescents. The adolescents in the study community represent intra-ethnic discourse, which is beyond this study. There is an ongoing debate among Sami and many indigenous communities worldwide regarding who is a genuine member of an indigenous community. Lack of recognition by one’s own group is generally detrimental to health and should be considered by all members of the community when discussing ethnicity.

## Appendix

Research themes and some selected questions.

### Time spent

What did you do yesterday from when you woke up to when you went to sleep?

Is this a normal day for you? Explain.

Was this day as you would have liked it to be?

If yes: Please explain what was good about it.

If no: Why? What would you have liked better?

### Life-history timeline

We would like to know about your life in all its detail.

We would like to hear about good times, tough times and how you got through the tough times.

There are some areas we will ask you about, but it is up to you to tell us what you think is most important.

Tell us about events you remember, your family and friends, school and anything else that you think has been important to you. We will meet to go over your life more than once, so we don’t sit here too long at one sitting.

Tell me about your life, starting with your earliest memory and continuing until now. (Ask questions that help the participant tell stories about his/her life, important relationships and events, and that help us understand who he or she is.)

Start drawing a life timeline. Explain that “The timeline is just to get us going. We can add to it as we go along. I will be noting things as you talk so that I can remember to follow up about different things.”

### Challenges/problems and how they are overcome

What are some challenges or problems you have faced?

The interviewer will pick one of these problems or challenges and ask: what helped you get through this?

Ask about another challenge or problem. “Tell me about another problem/challenge. What helped you get through this?”

What have you had to deal with lately – it could be big or small, but something that has happened in the last few weeks. How did you handle that?

If you had to come up with a few words to describe how you normally deal with problems, what would you say?

We’ve asked you a lot about your problems or challenges, and I wonder: what do you think are the biggest problems for youth in the community?

### Relationships

Sociogram (get full names of people and their relationship to the participant; e.g. aunt, uncle, cousin, friend, etc.)

Tell me about the people in your life. (Refer back to the sociogram to get names and the kinds of relationships they have with individuals.)

### School

What are the biggest problems young people have with school?

What about you?

How do you deal with such problems?

What would make school better?

Have you ever avoided going to school? Why?

What kinds of things have you learned in school that you think will help you in the future?

If the person is not attending school: why aren’t you in school? What would help you go back to school? What could we do more of to keep kids in school?

### Sami culture and language

Tell me about what being Sami means to you? Do you feel like a Sami? Do you speak the language?

What does it mean to you to be able to speak Sami? What do you appreciate of traditional Sami knowledge?

Is anybody teaching you about traditional knowledge? Can you be specific about the things you have learned?

When you go outside the community to big Norwegian cities, do you tell people you are Sami or do you try to hide it? Have you sometime felt ashamed by being a Sami?

Have you been proud of being a Sami?

What’s important to you about your traditional culture? Who do you learn about your culture from?

### Family

Tell me about your family.

How do members of your family spend their time? If you or someone else in your family stepped out of line, what would happen?

How often do you really talk to an adult – it could be a parent, aunt, uncle, grandparent – in your family?

What do you usually talk about?

### Youth in community

Tell me about the community.

What are the best things about growing up here?

What do you think would make it a better place to grow up?

### Future hopes and plans

Tell me what you think your future will be like.

What do you hope for your future? Has anyone talked with you about this?

Who do you know that has a life like the one you describe? Tell me about him or her.

What do you expect of your future? Has anyone talked with you about this? Please tell me more. Do you see yourself staying in the community or moving away? Why?

What should young people know in order to become adults around here?

### Health

What does good health mean to you?
